# Russell-Silver Syndrome With Growth Hormone Deficiency

**DOI:** 10.7759/cureus.60018

**Published:** 2024-05-10

**Authors:** Hiya Boro, Shinjan Patra, Kiran Kumar Pasam, Mazhar Dalvi, Vikash Bundela

**Affiliations:** 1 Endocrinology, Aadhar Health Institute, Hisar, IND; 2 Endocrinology, All India Institute of Medical Sciences, Nagpur, IND; 3 Endocrinology, Asian Institute of Gastroenterology, Hyderabad, IND; 4 Endocrinology, Mediclinic Al Noor Hospital, Abu Dhabi, ARE; 5 Gastroenterology, Aadhar Health Institute, Hisar, IND

**Keywords:** growth hormone stimulation test, growth hormone therapy, insulin-like growth factor 1, clinodactyly, insulin-like growth factor ii, growth hormone deficiency, russell-silver syndrome

## Abstract

Russell-Silver syndrome (RSS) is a rare genetic disorder characterized by intrauterine growth restriction (IUGR), postnatal growth failure, and distinctive dysmorphic features. We present a case of a four-year-old male presenting with a slow growth velocity with a history of IUGR and surgical interventions, exhibiting classic RSS features. Laboratory investigations revealed low insulin-like growth factor 1 (IGF-1) and low growth hormone (GH) levels on stimulation tests. Clinical exome sequencing revealed a de novo mutation in the insulin-like growth factor 2 *(IGF2)* gene. Additionally, a variant of uncertain significance in the *DHX37* gene was noted in the patient and the asymptomatic father. After genetic counseling, recombinant GH therapy was initiated. This case underscores the genetic complexity of RSS and highlights the importance of early diagnosis, genetic testing, and multidisciplinary management in optimizing outcomes for patients with RSS.

## Introduction

Russell-Silver syndrome (RSS) is a rare but clinically significant genetic disorder characterized by intrauterine growth restriction (IUGR), postnatal growth failure, and distinctive dysmorphic features including body asymmetry [1]. First described in 1953 by Russell and Silver [2,3], the syndrome has since been recognized as a complex condition with variable clinical manifestations and genetic underpinnings. RSS poses diagnostic challenges due to its phenotypic variability and overlap with other syndromes like Three M, IMAGe syndrome (intrauterine growth restriction, metaphyseal dysplasia, adrenal hypoplasia congenita, genital abnormalities), Bloom syndrome, Temple syndrome, fetal alcohol syndrome, and Beckwith-Wiedemann syndrome (BWS), necessitating a comprehensive approach to diagnosis and management.

In this case report, we describe a four-year-old male child who presented to us with distinctive dysmorphic features suggestive of RSS and growth failure.

## Case presentation

A four-year-old male child, born of non-consanguineous parentage, presented to the Endocrinology department at a hospital in north India, with a history of growth failure and distinctive dysmorphic features. The patient was the only child of his parents, and there was no similar history among any of the family members on either the maternal or paternal side. The perinatal history was notable for IUGR at term gestational age with a birth weight of 1.7 kg (- 4.0 standard deviation score (SDS)) and feeding difficulties at birth. There was no history of neonatal jaundice, hypoglycemia, or convulsions. The child had difficulty in passing urine and was diagnosed with left hydronephrosis due to obstruction of the pelvic-ureteric junction for which he underwent pyeloplasty. He also had trigonocephaly at birth for which he underwent cranial vault reconstruction.

**Figure 1:**
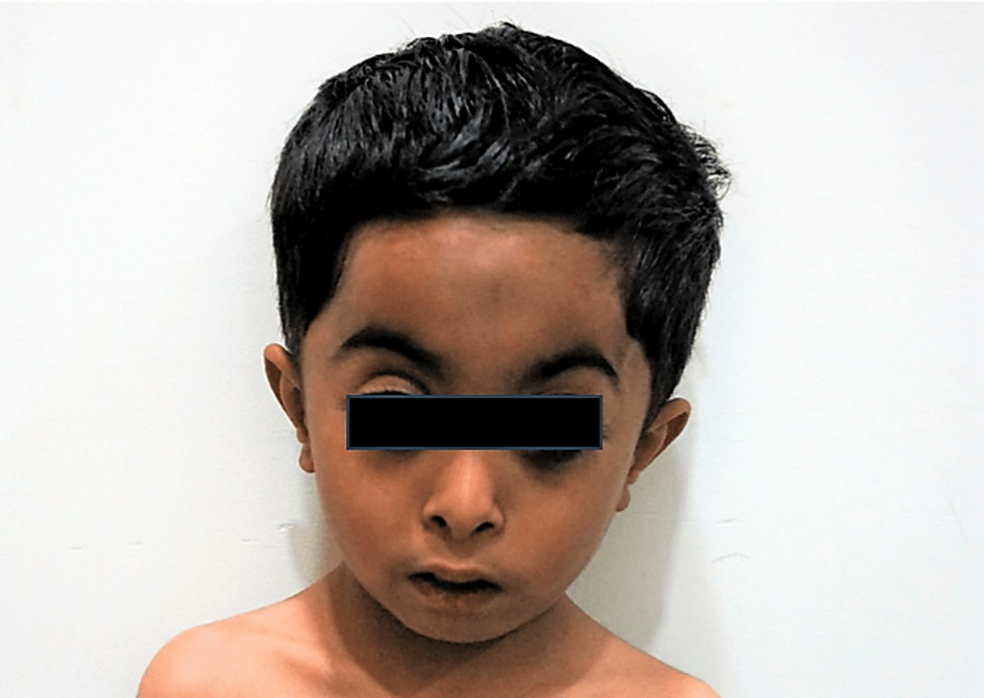
Clinical image of the patient with triangular facies, prominent forehead, high arched eyebrows, hypertelorism, and pinched nose

**Figure 2:**
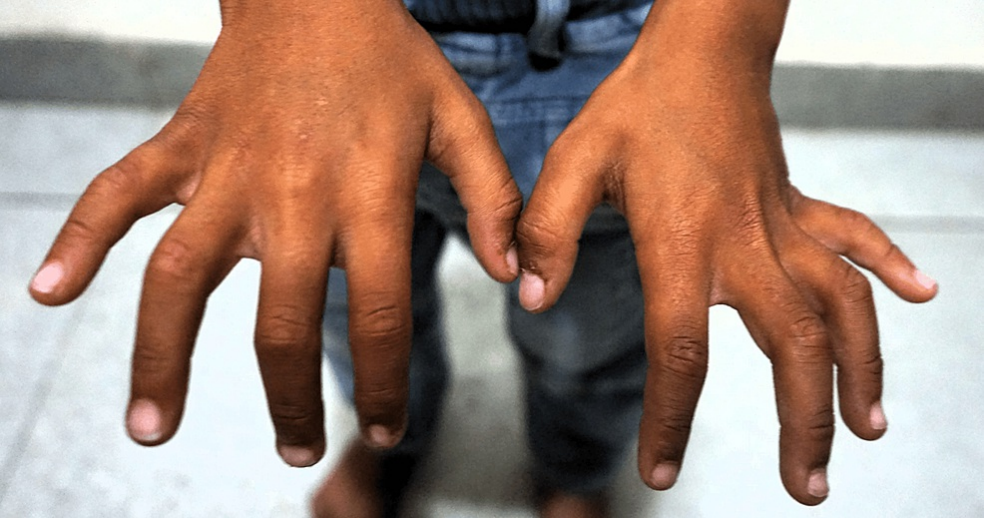
Clinodactyly of fingers

The current physical examination revealed distinctive features of RSS in the form of triangular facies, prominent forehead, high arched eyebrows, hypertelorism, pinched nose, depressed nasal bridge, microstomia, prominent incisors, retro micrognathia, and bilateral clinodactyly (Figures 1, 2). Notably, there was no apparent asymmetry in body proportions. Genital examination revealed normal findings, with bilaterally descended testes. Scars from previous surgeries were evident and were healthy and clean.

**Figure 3:**
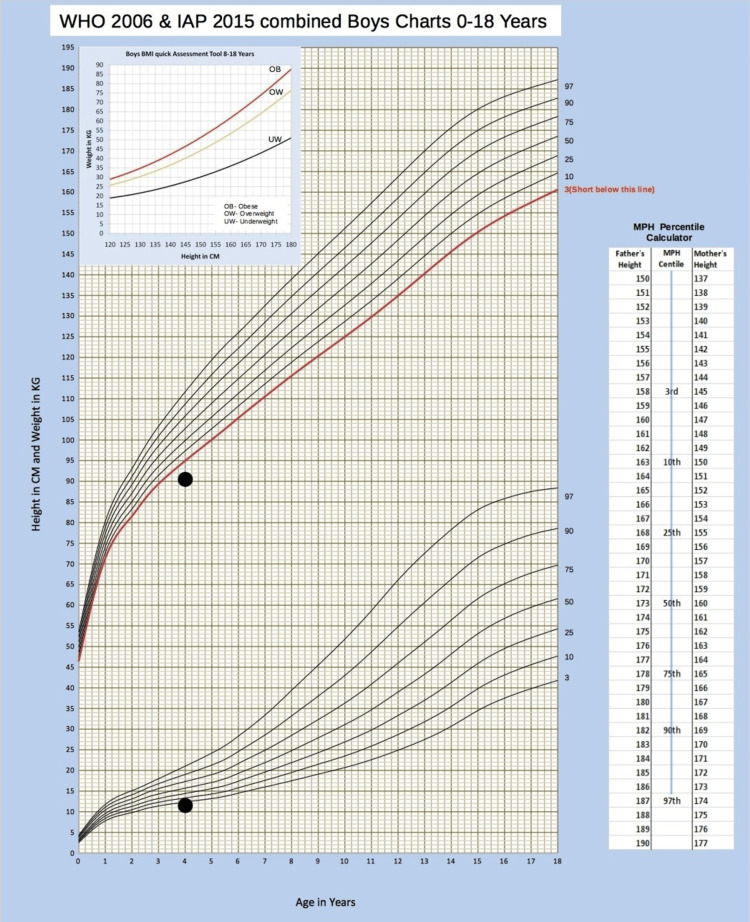
Growth chart of the patient Growth chart of the patient showing height and weight below 3rd centiles, as per the Indian Academy of Pediatrics growth chart for boys aged 0-18 years [4-6]. This image is published under a Creative Commons Licence

The current height was 90 cm (- 3.17 SDS) and the weight was 11 kg (- 3.05 SDS), both of which were below the 3rd centiles, as per the Indian Academy of Pediatrics growth chart for boys aged 0-18 years (Figure 3) [4-6]. Head circumference was 50 cm (0.39 SDS). There was no motor, speech, language, or intellectual impairment in the child. 

Laboratory investigations were notable for low levels of serum insulin-like growth factor-1 (IGF-1) at 24.6 ng/ml (normal range: 24.9-208.0 ng/ml), raising suspicion of growth hormone (GH) deficiency. For confirmation, a GH stimulation test with clonidine at a dose of 0.15 mg/m^2^ of body surface area was done and GH samples were collected at 0, 30, 60, 90, and 120 minutes. The results of the test are mentioned in Table 1. All GH values were less than 7.0 ng/ml, thus confirming GH deficiency [7]. 

**Table 1:** Growth hormone levels on the clonidine stimulation test

Timings (minutes)	Growth hormone (GH) levels (ng/ml)
0	2.91
30	5.69
60	6.17
90	2.36
120	3.04

Other pituitary hormones including serum thyroxine (T4), serum thyroid stimulating hormone, and morning cortisol were within normal limits. Complete hemogram, liver, and kidney function tests, tissue transglutaminase test for coeliac disease, and arterial blood gas analysis for renal tubular acidosis were within normal limits. The radiograph for bone age corresponded with the chronological age of four years. Contrast-enhanced magnetic resonance imaging (MRI) of the pituitary did not reveal any abnormality.

To elucidate the genetic basis of the patient's condition, clinical exome sequencing was performed, revealing an autosomal dominant heterozygous mutation in exon 2 of the insulin-like growth factor 2 *(IGF2)* gene at chromosome 11 that resulted in the amino acid substitution of serine for glycine at codon 34 (c.100G>A; p.Gly34Ser). Importantly, this variant was not detected in the asymptomatic parents, indicating a de novo mutation in the patient.

In addition to the *IGF2* gene mutation, the child also harbored an autosomal dominant heterozygous missense variant in exon 27 of the DEAH-box helicase 37 *(DHX37)* gene at chromosome 12 (c3434C>T; pPro1145Leu) that resulted in the amino acid substitution of leucine for proline at codon 1145. The same variant of uncertain significance was detected in a heterozygous state in the asymptomatic father of the patient.

Based on the clinical and genetic findings, a diagnosis of RSS was established. The patient was initiated on recombinant GH therapy at a dose of 0.3 mg/kg body weight/week in daily divided doses, with regular monitoring of auxological parameters (height, weight, head circumference) and serum IGF-1 levels to assess treatment response and adjust therapy as needed. Genetic counseling was provided to the parents of the child and the complexities of the disease and the need for life-long surveillance were explained in lucid language. At the time of writing this case report, the child has completed a month of GH therapy and there has been no apparent change in the anthropometric parameters.

## Discussion

RSS displays a broad spectrum of clinical manifestations, ranging from mild to severe growth restriction and dysmorphic features. The Netchine-Harbison Clinical Scoring System (NH-CSS) serves as a sensitive diagnostic tool for RSS [8]. Diagnosis is typically confirmed if an individual meets at least four NH-CSS clinical criteria, including a prominent forehead/frontal bossing and relative macrocephaly at birth, along with two additional findings such as being small for gestational age, post-natal growth failure, body asymmetry, and feeding difficulties, while other disorders have been ruled out. However, not all patients exhibit the full array of features, which can present diagnostic challenges, particularly in milder cases. Differential diagnoses such as Three M syndrome, IMAGe syndrome, Bloom syndrome, Temple syndrome, fetal alcohol syndrome, and BWS among others should be considered and ruled out [1]. Although these syndromes share certain similarities, they possess distinct features that differentiate them from RSS. Additionally, the genetic mutations associated with these syndromes differ from those observed in RSS. Table 2 delineates the genetic mutations, commonalities, and distinguishing characteristics of each syndrome in comparison to RSS.

**Table 2:** Differential diagnoses of RSS The table illustrates the genetic mutations, overlapping, and distinguishing features of various syndromes in comparison to RSS [1].

Disorder	Genetic mutations	Overlapping features with RSS	Distinguishing features from RSS
Three M syndrome	CCDC8, CUL7, OBSL1	Frontal bossing, macrocephaly, triangular facies, clinodactyly	Short neck, rib hypoplasia, pectus excavatum
IMAGe syndrome	CDKN1C	Frontal bossing, macrocephaly	Adrenal insufficiency, metaphyseal dysplasia
Bloom syndrome	BLM	Triangular facies, clinodactyly	Dolicocephaly, microcephaly
Temple syndrome	Maternal uniparental disomy 14, paternal chromosome 14 deletion, or loss of methylation at 14q32	Many features overlap with RSS	Distinguished by genetic testing
Fetal alcohol syndrome	Acquired disease	Small for gestational age, postnatal growth failure, 5^th^ finger clinodactyly	Microcephaly, hypoplastic philtrum, history of in-utero exposure to alcohol, short palpebral fissures
Beckwith-Wiedemann syndrome (BWS)	Imprinting defects in 11p15.5, CDKN1C	Macrosomia, body asymmetry, small for gestational age, neonatal hypoglycemia	Macroglossia, embryonal tumors, visceromegaly, kidney abnormalities

The genetic basis of RSS is complex and heterogeneous [9]. While most cases are sporadic, familial cases with autosomal dominant or imprinting defects have been reported. Genetic testing confirms the clinical diagnosis of RSS in about 60% of cases [1]. Epigenetic changes including hypomethylation of the imprinted control region 1 (ICR1) at chromosome 11 account for 35%-50% of cases, while maternal uniparental disomy (mUPD) of chromosome 7 causes 7%-10% of cases [1]. A few individuals with RSS have duplications, deletions, or translocations involving the imprinting centers at chromosomes 11 and 7 [1]. Rarely, pathogenic variants in genes like *CDKN1C*, *IGF2*, *PLAG1*, and *HMGA2* are found [1]. However, approximately 40% of individuals meeting clinical criteria for RSS have negative genetic testing results [1].

In our case, the discovery of a de novo mutation in the *IGF2 *gene delineates the significance of genetic mutations within the insulin signaling pathway in RSS pathogenesis. IGF-II plays a pivotal role in fetal growth, predominantly expressed from the paternal allele in the placenta during gestation [10,11]. Following birth, IGF-II production transitions to the liver. Reduced *IGF2* gene expression due to hypomethylation of ICR1 primarily affects prenatal growth. Notably, even in cases with normal serum IGF-II levels, tissue levels may remain deficient postnatally, impacting growth beyond birth as well [11].

In addition, the identification of a variant of uncertain significance in the *DHX37* gene highlights the genetic complexity of RSS and the potential role of additional genetic factors in disease manifestation. We conducted an extensive literature search to explore any potential association between the *DHX37* mutation and RSS. Unfortunately, our investigation did not uncover any evidence supporting such an association. Furthermore, the variant has not been documented in the 1000 Genomes and gnomAD databases. *DHX37* genetic mutation has been previously linked to 46 XY disorder of sex development (DSD), characterized by a spectrum of genital phenotypes ranging from predominantly female to predominantly male, with varying degrees of sex ambiguity depending on the duration of normal testicular function before the loss of testicular tissue [12]. Although cryptorchidism has been reported in cases of RSS [13], our patient did not present with any genital abnormalities. Further tests, such as in silico analysis, are warranted to ascertain whether the genetic mutation represents a pathogenic variant.

Short stature is a key feature of RSS [14]. Growth failure is progressive in childhood, with the mean final height, without GH treatment, being -3.6 SDS below the mean for normal children of the same age and sex. GH deficiency (GHD), along with abnormalities in nocturnal GH pulses, is common in RSS [14]. GHD is diagnosed using stimulation tests that involve administering substances that should normally trigger GH release such as insulin, arginine, levodopa (L-dopa), clonidine, glucagon, and growth hormone release hormone (GHRH) [15]. Blood samples are taken fasting, then after administering the agent, samples are taken every 30 minutes for 2-3 hours. The uniform threshold for diagnosing GHD remains a subject of debate, owing to variations in peak GH levels contingent upon the stimulus and assay employed [15]. Initially set at <2.5 ng/mL, the cutoff for GHD diagnosis was later revised upward due to the availability of recombinant human GH [14]. Presently, a cutoff of 10 ng/mL is predominantly utilized, although its reliability is questioned, leading to reduced specificity and the potential misclassification of normal children as GHD-positive. Some studies have proposed a lower threshold of <7 ng/mL for GHD diagnosis [7]. In our patient, all GH values obtained during clonidine stimulation were consistently below 7 ng/mL, strongly indicative of GHD [7].

Recombinant GH therapy has emerged as a cornerstone of treatment for RSS-associated growth failure [13]. By stimulating linear growth and improving body composition, GH therapy can significantly enhance the quality of life and long-term outcomes for patients with RSS. Regular monitoring of growth parameters, bone age, and biochemical markers such as serum IGF-1 levels is essential to optimize treatment efficacy and minimize potential adverse effects. However, RSS patients with loss of methylation at 11p15 may have high baseline IGF-1 levels, indicating some degree of IGF-1 resistance [13]. Comparative analysis of their IGF1 levels against age-appropriate norms often reveals values trending toward the upper end of the normal range [13]. Similarly, their IGF binding protein 3 (IGFBP3) levels frequently skew toward the higher spectrum. Upon administration of standard GH doses, IGF1 levels may substantially surpass the established reference range. Further investigations are warranted to elucidate optimal strategies for utilizing these biomarker levels in monitoring GH dosage adjustments in RSS patients exhibiting IGF1 resistance.

The management of RSS extends beyond GH therapy and includes nutritional support, physical therapy, and psychosocial interventions to address developmental delays and psychosocial issues [13]. Multidisciplinary collaboration involving pediatric endocrinologists, geneticists, nutritionists, and developmental specialists is crucial for comprehensive care and long-term management planning.

## Conclusions

In conclusion, RSS is a rare genetic disorder characterized by IUGR, postnatal growth failure, and distinctive dysmorphic features. Early recognition and diagnosis of RSS are paramount for initiating timely interventions, including growth hormone therapy, to optimize growth and developmental outcomes. Genetic testing, such as clinical exome sequencing, plays a crucial role in confirming the diagnosis and identifying underlying genetic abnormalities, as demonstrated in our case with mutations in the *IGF2* and *DHX37* genes.

Further research into the molecular mechanisms underlying RSS, genotype-phenotype correlations, and the contribution of additional genetic factors is needed to improve diagnostic accuracy, therapeutic efficacy, and overall prognosis for patients with this complex disease.
